# Genetic Markers of Insulin Resistance and Atherosclerosis in Type 2 Diabetes Mellitus Patients with Coronary Artery Disease

**DOI:** 10.3390/metabo13030427

**Published:** 2023-03-14

**Authors:** Sangeetha Perumalsamy, Hasniza Zaman Huri, Bashar Mudhaffar Abdullah, Othman Mazlan, Wan Azman Wan Ahmad, Shireene Ratna D. B. Vethakkan

**Affiliations:** 1 Department of Clinical Pharmacy and Pharmacy Practice, Faculty of Pharmacy, Universiti Malaya, Kuala Lumpur 50603, Malaysia; 2 Department of Medical Laboratories Techniques, Al-Rafidain University College, Baghdad 46036, Iraq; 3 Cardiology Unit, Department of Medicine, Faculty of Medicine, Universiti Malaya, Kuala Lumpur 50603, Malaysia; 4 Endocrinology Unit, Department of Medicine, Faculty of Medicine, Universiti Malaya, Kuala Lumpur 50603, Malaysia

**Keywords:** insulin resistance, type 2 diabetes mellitus, atherosclerosis, endothelial dysfunction, coronary artery disease, genetic markers, single nucleotide polymorphisms

## Abstract

Type 2 diabetes mellitus (T2DM) is characterized by impaired insulin secretion on a background of insulin resistance (IR). IR and T2DM are associated with atherosclerotic coronary artery disease (CAD). The mechanisms of IR and atherosclerosis are known to share similar genetic and environmental roots. Endothelial dysfunction (ED) detected at the earliest stages of IR might be the origin of atherosclerosis progression. ED influences the secretion of pro-inflammatory cytokines and their encoding genes. The genes and their single nucleotide polymorphisms (SNPs) act as potential genetic markers of IR and atherosclerosis. This review focuses on the link between IR, T2DM, atherosclerosis, CAD, and the potential genetic markers *CHI3L1*, *CD36*, *LEPR*, *RETN*, *IL-18*, *RBP-4*, and *RARRES2* genes.

## 1. Background

Type 2 diabetes mellitus (T2DM) accounts for over 90 per cent of all patients with diabetes [1]. T2DM shares several risk factors in common with coronary artery diseases (CAD), such as aging, hypertension, dyslipidemia, obesity, lack of physical activity, genetics, and stress. In addition, an increase in the prevalence of diabetes indirectly escalates the risk of CAD [2]. T2DM is primarily caused by insulin resistance (IR), in which insulin cannot promote glucose uptake in skeletal muscle and adipose tissue and suppress hepatic glucose output [3,4].

Endothelial dysfunction (ED), the failure of endothelium to maintain vascular homeostasis, is present at the early stages of IR. It may be the origin of the initiation and progression of atherosclerosis [5]. Atherosclerosis that affects the coronary arteries can cause coronary artery disease (CAD) [6]. CAD is a type of cardiovascular disease (CVD) that is often asymptomatic in T2DM patients until the onset of myocardial infarction (MI) or sudden cardiac death [7].

IR may be associated with ED via various mechanisms such as disturbances of the subcellular signaling pathways that involve insulin action and nitric oxide (NO) production, oxidative stress, endothelin, the secretion of hormones and cytokines, as well as the renin–angiotensin–aldosterone system [8]. Changes in the subcellular signaling pathways convert the anti-atherogenic property of NO into pro-atherogenic and cause the development of atherosclerosis and subsequently CAD [9].

ED has been linked to several factors such as diabetes, hypertension, smoking, a high-fat diet, as well genetic factors [10]. The genetic factor of ED involves the dysregulation of the endothelial NO synthase (eNOS) gene followed by disruption of the endothelial vascular homeostasis [8]. It has been reported that the polymorphism caused by four or five repeats of a 27-base-pair sequence in intron 4 of the eNOS gene is associated with the risk of CAD [11]. Another study of the eNOS gene also found that intron 4 polymorphism was associated with T2DM [12]. Thus, the genetic factor of ED is linked to both T2DM and CAD [13].

The eNOS gene expression pathways involve the activation of the pro-inflammatory markers related to IR, atherosclerotic CAD and T2DM [14]. These genes and their single-nucleotide polymorphisms (SNPs) may act as the possible identifiers of IR and atherosclerosis in T2DM patients with CAD. This review focuses on the potential genetic markers of IR and atherosclerosis in T2DM with CAD, and identification of these potential genetic markers may further enhance the optimization of glycemic control and T2DM management.

## 2. How Does IR Result in T2DM?

Insulin is a peptide hormone secreted by the β cells of the pancreatic islets of Langerhans. It maintains normal blood glucose levels by facilitating cellular glucose uptake, regulating carbohydrate, lipid, and protein metabolism, and promoting cell division and growth through its mitogenic effects [15]. Insulin secretion may be influenced by alterations in synthesis at the level of gene transcription, translation, and post-translational modification in the Golgi among other factors influencing insulin release from the secretory granules [15,16].

The actions of insulin are influenced by the interplay of other hormones such as growth hormone, IGF-1, glucagon, glucocorticoids, and catecholamines [17]. Excessive hormone production might have contributed to IR in some circumstances, though com-promised insulin signaling is thought to have a greater role at the cellular level [18]. As such, IR can be considered as a state of chronic, low-level inflammation [19]. Several states or mechanisms result in IR, such as fatty acid-induced IR [20] and lipid accumulation in skeletal muscle and liver [21].

**Figure 1:**
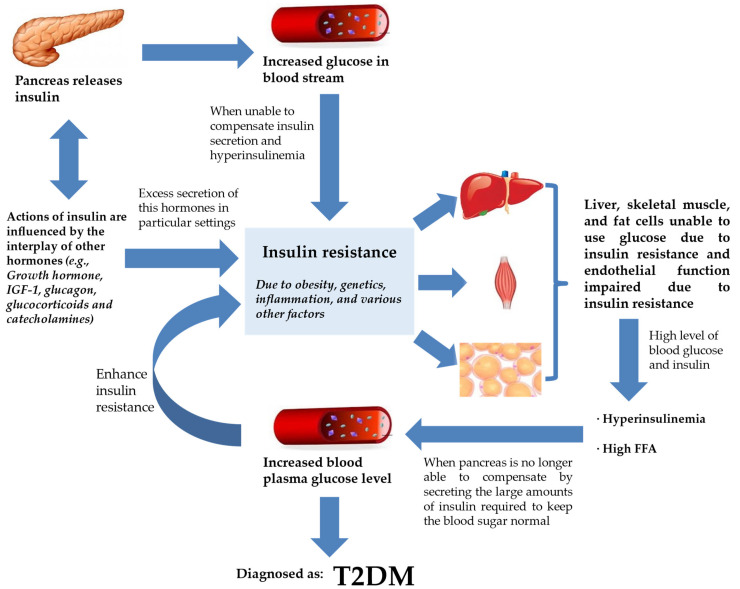
IR and T2DM. Insulin secretion by the pancreas is influenced by the interplay of various hormones (hormone, IGF-1, glucagon, glucocorticoids, and catecholamines) in order to regulate glucose levels in the bloodstream. However, insulin failure to manage blood glucose will lead to the progression of IR. IR will compromise endothelial function, rendering liver, skeletal muscle, and fat cells unable to use glucose. This worsens hyperinsulinemia, and the pancreas is already incapable of keeping normal blood glucose despite secreting large amounts of insulin. Blood plasma glucose levels keep increasing and the feedback loop enhances IR further. At this stage, the patient is diagnosed with T2DM. Abbreviations: ED, endothelial dysfunction; FFA, free fatty acid; IGF-1, insulin-like growth factor 1; IR, insulin resistance; T2DM, type 2 diabetes mellitus.

The beginning of IR frequently results in insulin paucity and a gradually dwindling blood glucose regulation, hyperinsulinemia, and increased levels of free fatty acid (FFA) circulation [22,23]. The circulating FFAs are the main substrate for production of hepatic triglycerides in the form of very low-density lipoprotein (VLDL) [24,25,26]. These changes are trailed by a subsequent decline in plasma glucose control, which manifests as elevated fasting plasma glucose levels with intermittent and persistent hyperglycemia leading to a diagnosis of T2DM (Figure 1) [27]. Therefore, in terms of pathogenesis, glucolipotoxicity is stated as an essential determinant of T2DM. Glucolipotoxicity is the combination of glucotoxicity (hyperglycemia or elevated blood glucose levels) and lipotoxicity (high lipid levels, especially FFAs) [28].

From Figure 1, T2DM can be seen as end-stage IR in a genetically susceptible individual. The effects of insulin, insulin shortage, and IR, however, vary depending on the physiological function of the tissues and organs and their reliance on insulin for metabolic activities [29]. The direct and indirect effects in tissues sensitive to insulin are linked to glucose homeostasis in the liver, skeletal muscle, and fat tissues [30]. Another site of insulin action and the manifestation of IR and T2DM is the endothelium [31]. With ED being observed at the early phase of atherosclerosis, the vascular endothelial cells may play critical roles in various facets of cardiovascular biology [8].

IR is strongly prevalent in the pathogenesis of T2DM [32]. A study in the United Kingdom found that almost 8% of 6500 participants were diagnosed with diabetes throughout the course of the study’s 10 years of follow-up [33]. There was a significant decline in insulin sensitivity in the five years before the diabetes diagnosis in comparison to those who were not diagnosed with diabetes.

## 3. Atherosclerosis and Its Relationship with IR, T2DM, and CAD

T2DM and atherosclerotic CVD share many common factors. In T2DM, inflammatory processes play a part in the cause of atherosclerotic CVD [34]. The markers of inflammation predict CAD and the levels of markers are raised in patients with T2DM [35]. ED is considered as an early marker for atherosclerosis, and it plays a prominent role in the development of IR and T2DM [5]. IR and impaired insulin secretion are central to the pathogenesis of T2DM, but it is unclear how these abnormalities are related to accelerated atherosclerosis [36]. The precise mechanisms for the susceptibility and advancement of atherosclerosis in patients with T2DM are undetermined.

Yet, there are many indications linking lipoproteins with atherosclerosis [37]. Lipoproteins are the combinations of fats and protein. The lipoproteins are proved to play an important role in atherosclerosis, and they interact with the artery wall to trigger the chain of events that leads to atherosclerosis. Low-density lipoprotein (LDL) is the most common lipoprotein that is associated with atherosclerosis, but other lipoproteins such as VLDL may also be atherogenic. An important component of these atherogenic lipoproteins is Apolipoprotein B (Apo B) that promotes the accumulation of LDL in the intima initiates atherosclerosis [38]. This is mediated by increased endothelial permeability and raised intimal retention of LDL [39]. Moreover, diabetes (T1DM and T2DM) is associated with increased hepatic production of triglyceride-rich lipoproteins, which leads to increased formation of atherogenic VLDL [40]. The atherogenic index is defined as the ratio of LDL-C (low-density lipoprotein cholesterol) to HDL-C (high-density lipoprotein cholesterol). In one study, researchers discovered that hyperlipidemic rats had a higher atherogenic ratio (8.06 mg/dL) than the control group (1.09 mg/dL) [41].

Metabolic syndrome, pre-diabetes, and T2DM, which all co-segregate with IR, accelerate the progression of atherosclerosis and the consequential disease [34].

**Figure 2:**
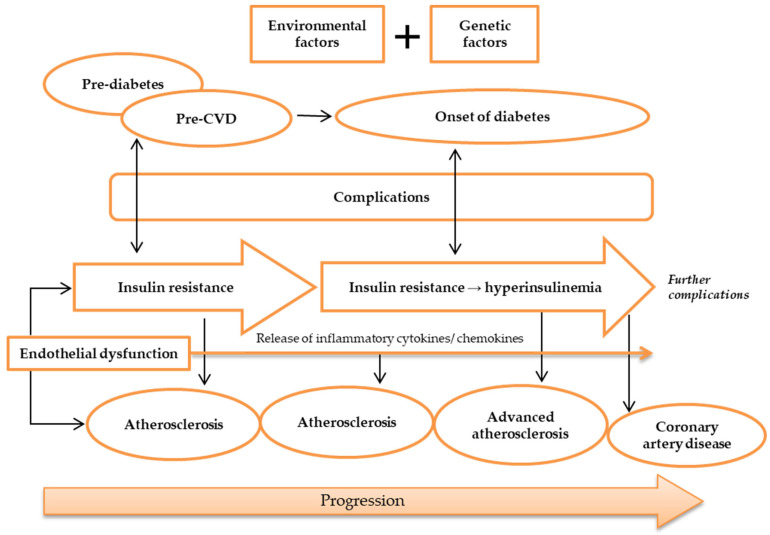
Pathogenesis of atherosclerosis. Atherosclerosis may be caused by environmental and genetic factors. The onset of atherosclerosis is during ED initial occurrence, which is also associated with the onset of IR. These incidences reflect pre-diabetes and pre-CVD phases, and symptoms of complications may appear. Ongoing atherosclerosis development is incessantly supported by ED, linked to the release and regulation of inflammatory cytokines and chemokines, as well as IR, which develops into hyperinsulinemia. At this stage, patients may have developed advanced atherosclerosis and diabetes, while exhibiting evident complications. Further ED, IR, and atherosclerosis advancement will result in more apparent complications and CAD development. Abbreviations: CAD, coronary artery disease; CVD, cardiovascular disease; ED, endothelial dysfunction; IR, insulin resistance.

Silent atherosclerosis and cardiovascular complications start to commence during the pre-diabetic period in genetically susceptible people [42]. During the onset of diabetes, hyperglycemia and hyperinsulinemia are present in IR and lead to acceleration of atherosclerosis and CAD (Figure 2) [43,44]. Still, despite the fact that diabetic patients developed severe lesion formation, patients with and without diabetes often have identical atherosclerotic lesions [36]. Areas of necrotic lesions accumulate lipid, cholesterol crystals, and inflammatory cells, which leads to thickening of the arterial wall. Complications can arise from erosion or calcification, causing a thrombus to form and obstruct the lumen, resulting in health defects such as CAD [8,44].

Moreover, genetic factors have been investigated to modulate atherosclerosis development. Candidate gene and linkage analysis studies have failed to identify previously unknown pathways in the pathogenesis of atherosclerosis [45,46]. The publication of the HapMap has made possible genome-wide association studies aimed at probing the pathogenesis of atherosclerosis. Genome-wide association studies have reproducibly identified several loci involved in the pathogenesis of atherosclerosis, and most of the identified genes are newly implicated in the disease process. APOE- or LDLR-deficient mice are widely used models to study the pathogenesis of atherosclerosis.

## 4. How Do IR and Atherosclerosis Link Genetically?

Patients with T2DM are at higher risk of developing CAD. It has previously been proposed that diabetes and atherosclerotic CVD share the same genetic and environmental roots [47]. Although IR has been postulated to involve many mechanisms in developing other diseases, the major process in developing atherosclerotic CAD from IR is ED [5]. Endothelium-derived NO regulates endothelial function. It is synthesized from L-arginine by NO synthase encoded by the endothelial NO synthase (eNOS) gene, which is mapped on chromosome 7 (7q35-7q36) [5,48]. When IR affects cells, there is an increase in oxidative stress and the protein kinase C and receptor for advanced glycation end products (RAGE) will be activated [49]. This will be followed by several changes in the endothelium, primarily compromised eNOS gene activity that decreases NO bioavailability and ultimately causes ED [48].

ED results in vasoconstriction, inflammation, and thrombosis that may contribute to the development of the atherosclerosis [50]. At the same time, ED will increase the IR condition, mainly the inflammatory process [34]. The inflammatory process involves the release of pro-inflammatory cytokines and chemokines that cause IR and the development of atherosclerosis [51]. The pro-inflammatory cytokines and chemokines are encoded by genes that may be associated with both IR and atherosclerosis [52]. Expressing these genes may serve as the potential genetic markers of IR and atherosclerosis.

Various studies have documented the association of genetic markers with IR and atherosclerosis. The common genetic markers of IR are IRSs genes, *SLC2A4* gene, *PTP1B* gene, *LEPR* gene, and *RETN* gene. *CHI3L1* [53], *CD36* [54], *IL-18* [55], and *RARRES2* [56,57] genes have also been reported to be associated with IR, whereas *APOA1*, *CD36*, *LEP*, *FN1*, and *CETP* genes were found to be associated with atherosclerosis. Gong et al. [58] found that the expression of *CHI3L1* was strongly connected with atherosclerotic risk factors and the severity of CAD. Nonetheless, the extent of the impact of genetic markers on IR and atherosclerosis is not fully understood.

**Figure 3:**
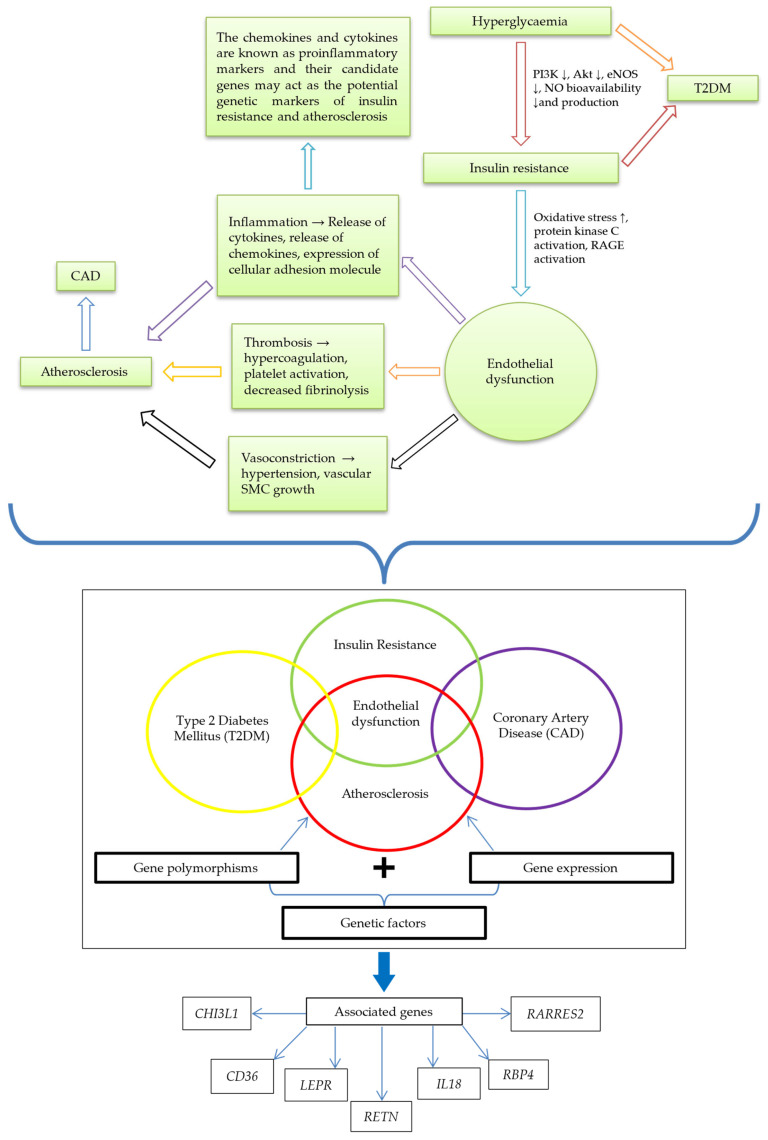
The link between IR and atherosclerosis; and potential genetic markers of IR and atherosclerosis. Hyperglycemia leads to IR, indicated by diminishing PI3K, Akt, eNOS, and NO bioavailability and production. IR causes ED, signified by increased oxidative stress and activation of protein kinase C and RAGE. Through inflammation, thrombosis, and vasoconstriction, ED brings about atherosclerosis and eventual CAD development. The relationship of genetic factors (gene polymorphisms and expression) highlighted ED in a central position among IR, CAD, atherosclerosis, and T2DM interplay. The associated genes are CHI3L1, CD36, LEPR, RETN, IL-18, RBP4, and RARRES2. Abbreviations: ↓, reduction; AKT, protein kinase B; CAD, coronary artery disease; CD36, cluster of differentiation 36; CHI3L1, chitinase-3 like-protein-1; ED, endothelial dysfunction; eNOS, endothelial nitric oxide synthase; IL-18, interleukin 18; IR, insulin resistance; LEPR: leptin receptor; PI3K, phosphoinositide 3-kinase; RAGE, the receptor for advanced glycation end products; RARRES2, retinoic acid receptor responder 2; RBP-4, retinol-binding protein 4; RETN, resistin.

We have identified seven genes (*CHI3L1*, *CD36*, *LEPR*, *RETN*, *IL-18*, *RBP-4*, and *RARRES2*) that may be associated with IR and atherosclerosis as having possible evidence based on the disease pathogenesis of ED and inflammation. The pathway that links ED with IR and atherosclerosis is summarized in Figure 3.

## 5. Genes and SNPs That May Be Associated with IR and Atherosclerosis in T2DM Patients with CAD

Identifying candidate gene polymorphisms begins with identifying risk variants and candidate SNPs associated with IR and atherosclerosis. The gene polymorphisms are typically called SNPs [59] and are the most common type of genetic variation among people. SNPs are single-nucleotide substitutions of one base for another that occur in more than one per cent of the general population [60]. Each SNP represents a difference in a single DNA building block, called a nucleotide. Reliable SNPs can be predictive markers that enable informed decisions about various aspects of healthcare, such as precise disease identification, as well as drugs’ efficacy, and negative feedbacks [61,62]. Even though several studies have reported an association of functional or position candidates, only some SNPs have been potentially associated with IR and atherosclerosis in T2DM patients with CAD (Figure 3 and Table 1). Following are the potential genes and their SNPs:

### 5.1. CHI3L1 Gene

The chitinase-3 like-protein-1 (*CHI3L1*) gene encodes YKL-40 (human cartilage glycoprotein-39) located on chromosome 1q31–1q32 [76]. YKL-40 is secreted by macrophages within the atherosclerotic plaques and is involved in inflammatory processes [77]. Patients with T2DM have elevated circulating YKL-40, parallel with their IR level [78]. YKL-40 is linked with all-cause mortality, including in patients with stable CAD [63]. The genetic variation in *CHI3L1* is strongly associated with YKL-40 levels [63,64]. The normal function of *CHI3L1* is catalyzing the hydrolysis of chitin and may also play a part in tissue remodelling and cells’ response to changes in their environment. Besides IR and atherosclerosis, *CHI3L1* is also involved in inflammatory processes [79].

Several SNPs of *CHI3L1* correlate with IR and CAD. Among the SNPs, rs946263 of *CHI3L1* is the most prominent that has been studied [63,64,80]. This SNP influences YKL-40 serum levels and low-density lipoprotein (LDL) levels in healthy individuals and patients with various inflammatory diseases such as CAD [63]. The *CHI3L1* level has been reported to be upregulated in patients with IR, T2DM, and CVD [81]. In a study among 290 Koreans, a significant association was shown between rs946263 and LDL serum levels, a major risk factor for the development of atherosclerosis [63]. The SNP rs946263 has also been studied in diabetes, but the associations were not fully elucidated [63,64]. Thus, rs946263 might be associated with both IR and atherosclerosis.

### 5.2. CD36 Genes

The *CD36* gene is located on chromosome 7q11.2 and is encoded by 15 exons [82,83,84]. CD36 is an 88-kD membrane glycoprotein categorized as a class B scavenger cell surface receptor that mediates internalization of oxidized low-density lipoprotein (Ox-LDL) leading to the formation of macrophage foam cells [85]. It presents on the surface of platelets, monocytes or macrophages, and endothelial and smooth muscle cells [86]. *CD36* serves as a candidate gene for impaired fatty acid metabolism, glucose intolerance, arterial hypertension, atherosclerosis, and numerous cardiovascular diseases [83,87] as well as Alzheimer’s disease [88] and malaria [89,90], and may be imperative in the pathogenesis of human IR syndromes. Deficiency of *CD36* is related to phenotypic expression of metabolic syndrome, which is commonly connected to atherosclerotic CVD, resulting in raised levels of glucose and thus contributing to T2DM [85]. In some studies, the *CD36* SNP, rs1761667, was correlated with T2DM [66,67]. This SNP has also been used in identifying cardiovascular events such as atherosclerosis [67,91]. In a study conducted among a Sohag population in Egypt, it was found that the AG genotype of the rs1761667 polymorphism in the *CD36* gene may have participated in CAD pathogenesis, body mass index (BMI) increase, and T2DM [67]. So, this SNP most probably will associate with both IR and atherosclerosis.

### 5.3. LEPR Gene

*LEPR* gene is located on chromosome 1p31, and it encodes leptin receptor (LepR) [92]. Leptin is synthesized in adipose tissue function to regulate appetite and body temperature [93]. Previously, leptin was considered an anti-obesity hormone, and later it was proposed to protect non-adipose tissues (e.g., liver, endocrine pancreas, heart) from lipotoxicity [94]. Leptin therapy has been reported to improve metabolic, glucose, and lipid imbalances in T2DM patients [95]. Meanwhile, studies on *LEPR* gene have associated it with MetS parameters such as IR [96], cardiovascular diseases [97], and hypertension [98]. Changes in the expression of leptin and *LEPR* due to genetic and environmental factors may lead to dysfunction of the leptin system, disturbances in energy balance, weight gain, and risk for developing T2DM or atherosclerosis [99]. The SNPs in *LEPR* have been reported to be associated with plasma LepR levels at the genome-wide significance level [100,101]. The rs1137100 SNP in the *LEPR* gene was independently associated with early atherosclerosis [68] and some risk factors of IR [102]. Lys109Arg of *LEPR* (rs1137100) has been shown to associate with some CVD risk factors [103]. Thus, this SNP may have a higher chance of association with IR and atherosclerosis.

### 5.4. RETN Genes

*RETN* encodes resistin and is located in chromosome 19p13.3 [104]. Resistin is cysteine-rich with 108 amino acid residues and is expressed largely in immune cells such as monocytes, macrophages, and neutrophils. Meanwhile, associations of *RETN* with plasma resistin levels, T2DM, and related metabolic traits have varied [105]. The blood circulating levels of resistin have been shown to be upregulated in subjects with IR, hypertension, T2DM, and CAD [106,107,108]. Additionally, *RETN*’s SNP rs3745367 has been implicated in cardiovascular disease, resistin levels, fasting glucose levels, and diabetic incidence [109,110,111,112], while rs1862513 was associated with IR and T2DM [111,113]. Hence, rs3745367 and rs1862513 might be potential markers for IR and atherosclerosis.

### 5.5. IL-18 Genes

*IL-18* gene is located on chromosome 11q22.2–q22.3 and contains numerous SNPs in the promoter region [114,115]. It encodes proinflammatory cytokine interleukin-18 (IL-18), which is central to the inflammatory chain reaction. The *IL-18* gene variations in the promoter region can influence IL-18 production and activity [116,117]. In patients with known CAD, circulating IL-18 levels and *IL-18* gene polymorphisms were associated with future cardiovascular mortality [118,119]. SNP rs1834481 within the *IL-18* gene was associated with IL-18 levels and IR [55]. The rs1834481 of *IL-18* has had reported effects on BMI in T2DM [120] and in subjects with CAD [72]. Polymorphism in *IL-18* has been associated with IL-18 levels in CAD patients with T2DM [121]. Therefore, *IL-18* SNPs can be the markers for IR and atherosclerosis.

### 5.6. RBP-4 Genes

The *RBP-4* gene is located on chromosome 10q23–q24 [74]. It encodes RBP-4 and is identified to link obesity with its comorbidities, especially IR and T2DM [122]. *RBP-4* gene expression in visceral adipose tissue is the most probable source for elevated RBP-4 serum concentrations in patients with increased visceral fat mass and T2DM. It contributes to the development of IR [51]. A study on Han Chinese found the *RBP-4* gene to be associated with CAD [74]. In addition to IR and T2DM links, the A/A genotype at the *RBP-4* rs7094671 locus also links with CAD in the population.

### 5.7. RARRES2 Gene

*RARRES2*, the encoding gene of chemerin, is located on chromosome 7q36.1. Chemerin is potentially involved in regulating immune responses at inflammation and tissue injury sites [123]. Chemerin, described as being secreted from mature adipocytes, has elevated circulating levels in human plasma parallel with obesity progression [124]. Patients with T2DM were investigated to present significantly higher chemerin values than controls [125]. The accumulation of chemerin in an atherosclerotic lesion contributes to atherosclerosis [126]. In addition, the severity of coronary atherosclerosis is found to be positively correlated with the level of *RARRES2* mRNA [127]. Furthermore, the bovine RARRES2 gene polymorphisms have been linked to T2DM [128]. SNP rs17173608 can be used as genetic determinants of IR [56,129]. The *RARRES2* variant rs17173608 was also associated with chemerin concentration and CAD [57,129]. In short, this *RARRES2* SNP might be the marker associated with IR and atherosclerosis.

## 6. Executive Summary

### 6.1. Genetic Markers and Disease

Genetic markers study the relationship between T2DM complications (IR and atherosclerosis) and their genetic cause. They are the variations or polymorphisms that can be observed. The studies that have been conducted thus far address the genetic polymorphisms of IR or atherosclerosis separately. Thus, future studies are crucial to investigate the association of potential mutual genetic polymorphisms with IR and atherosclerosis.

### 6.2. Mechanisms Involved in the Identification of Genetic Markers

The exact mechanisms for the increased susceptibility and progression of atherosclerosis in patients with diabetes are unknown. Still, ED is the common key event in IR and atherosclerosis progression. ED involves an inflammatory process. The inflammatory process involves the release of pro-inflammatory cytokines and chemokines encoded by genes that may be associated with IR and atherosclerosis. The encoding genes may serve as potential genetic markers.

### 6.3. Association of Potential Genetic Markers with IR and Atherosclerosis

Gene polymorphisms *CHI3L1* (rs946263), *CD36* (rs1761667), *LEPR* (rs1137100), *RETN* (rs1862513 and rs3745367), *IL-18* (rs1834481), *RBP-4* (rs7094671), and *RARRES2* (rs17173608) have the great potential to associate with IR and atherosclerosis in T2DM patients with CAD, as having possible evidence based on data from previous studies and the disease pathogenesis.

## 7. Conclusions

T2DM and CAD share several common risk factors such as aging, hypertension, dyslipidemia, obesity, lack of physical activity, genetics, and stress. IR is the main culprit contributing to T2DM and atherosclerotic CAD development. IR and atherosclerosis share the same genetic basis. ED and inflammation (involving pro-inflammatory markers) link IR and atherosclerosis. The pro-inflammatory markers’ encoding genes may be the potential genetic markers of IR and atherosclerosis in T2DM patients with CAD. This review identified *CHI3L1*, *CD36*, *LEPR*, *RETN*, *IL-18*, *RBP-4,* and *RARRES2* genes as the potential genetic markers of IR and atherosclerosis in T2DM patients with CAD.

## 8. Future Perspectives

Previous studies outlined genetic markers of IR and atherosclerosis separately in T2DM patients with CAD. The findings of this study offer an opportunity to improve management in T2DM patients with CAD by identifying new common genetic markers for IR and atherosclerosis. This could be accomplished by analyzing the diseases’ risk factors using specific SNPs linked to IR and atherosclerosis. SNPs identification methods such as TaqMan SNP are effective in genotyping the SNPs, and it has a high throughput and an accuracy of 99.9% and has long been regarded as the gold standard in qPCR. As a result, the data obtained from the genetic variations analysis will be consistent, precise, and reliable. This research will comprehend the genetics of pro-inflammatory markers (released secondary to ED) that may serve IR and atherosclerosis pathogenesis and provide novel insights into glycemic control and the progression of atherosclerosis in T2DM patients with CAD. Integrating genetic variation into clinical variables would be an added value for optimization, leading to cost-savvy effects in managing CAD in T2DM patients. The findings of this study pave the way for researchers to investigate further the functional variants in the coding regions of the candidate genes, as well as the relationship between IR, atherosclerosis, T2DM, CAD, and their risk factors. This will also spark future research if genetic variations in the ED pathway are linked to the risk of IR and atherosclerosis in T2DM patients in their respective populations.

## Figures and Tables

**Table 1:** Summarization of gene and SNPs associated with IR and atherosclerosis.

Gene	SNP ID	Position	Parameter Association	Population	N	*p*-Value	Ref
*CHI3L1*	rs946263	−9639 C > G	G-allele was nominally found to be associated with T2DM	Danish	9438	0.027	[63]
			Was associated with serum Ykl-40 levels	Danish	6784	<0.0001	[64]
		−2122 C > T	Was associated with LDL level (cause of atherosclerosis)	Korean	290	0.005	[65]
			Was associated with a significantly increased CHI3L1 mRNA level in peripheral blood cells and elevated nuclear factor binding			0.008	
*CD36*	rs1761667	−31118 G > A	Was associated with T2DM	North Indian	400	<0.001	[66]
			Was associated with lipid profile			<0.001	
			Was associated with LDL			<0.05	
			Was associated with VLDL			0.029	
		A > G	CAD patients with an AG genotype had higher plasma levels of LDL	Egyptian	100	0.046	[67]
*LEPR*	rs1137100	109 T > A	Was independently associated with early atherosclerosis	Finnish	526	0.042	[68]
			Was associated with high total cholesterol			0.005	
			Was associated with insulin levels				
*RETN*	rs1862513	−420 C > G	Was associated with resistin levels in DM patients	Japanese	198	2.9 × 10^−7^	[69]
			Was associated with HbA1c levels				
			Was associated with total cholesterol levels (CAD marker)	Pakistani	350	<0.0001	[70]
			Was associated with LDL levels (CAD marker)			0.0067	
			Was associated with resistin levels			0.0009	
			Was associated with hs-CRP levels (CAD marker)			<0.0001	
	rs3745367	+299 G > A	Significantly associated with T2DM	Thai	95	0.004	[71]
			Associated with total cholesterol levels	Pakistani		<0.0001	[70]
			Associated with LDL levels			0.0153	
			Associated with resistin levels			<0.0001	
			Associated with hs-CRP levels			<0.0001	
*IL-18*	rs1834481	G > C	Associated with CAD/ MI risk	European	3202	0.0021	[72]
			Associated with IL-18 levels	European	200	<0.005	[73]
			Associated with glucose levels			<0.005	
*RBP-4*	rs7094671	G > A	Associated with CAD	Chinese	392	<0.0001	[74]
		+5169 C > T	Associated with T2DM	Mongolian	281	<0.005	[75]
*RARRES2*	rs17173608	T > G	Associated with risk of metabolic syndrome (IR, high LDL)	Iranian	300	0.012	[57]

## Abbreviations

AKT: protein kinase B; APOA1: apolipoprotein A-I; APOE: apolipoprotein E; CAD: coronary artery disease; CD36: cluster of differentiation 36; CETP: cholesteryl ester transfer protein; CHI3L1: chitinase-3 like-protein-1; CVD: cardiovascular disease; ED: endothelial dysfunction; eNOS: endothelial nitric oxide synthase; FFA: free fatty acid; FN1: fibronectin 1; Hs-CRP: high-sensitivity C-reactive protein; IGF-1: insulin-like growth factor 1; IL-18: interleukin 18; IR: insulin resistance; LDL: low-density lipoprotein; LDLR: low-density lipoprotein receptor; LEPR: leptin receptor; MI: myocardial infarction; NO: nitric oxide; Ox-LDL: oxidized low-density lipoprotein; PI3K: phosphoinositide 3-kinase; PTP1B: phospho-tyrosine protein phosphatase 1B; RAGE: receptor for advanced glycation end products; RARRES2: retinoic acid receptor responder 2; RBP-4: retinol-binding protein 4; RETN: resistin; SLC2A4: solute carrier family 2 member 4; SMC: smooth muscle cells; SNPs: single-nucleotide polymorphisms; T2DM: type 2 diabetes mellitus; VLDL: very low-density lipoprotein.
